# Verification and Validation of Lower Body Negative Pressure as a Non-Invasive Bioengineering Tool for Testing Technologies for Monitoring Human Hemorrhage

**DOI:** 10.3390/bioengineering10101226

**Published:** 2023-10-20

**Authors:** Victor A. Convertino, Eric J. Snider, Sofia I. Hernandez-Torres, James P. Collier, Samantha K. Eaton, David R. Holmes, Clifton R. Haider, Jose Salinas

**Affiliations:** 1 Battlefield Health & Trauma Center for Human Integrative Physiology, US Army Institute of Surgical Research, JBSA Fort Sam Houston, San Antonio, TX 78234, USA; 2 Department of Medicine, Uniformed Services University of the Health Sciences, Bethesda, MD 20814, USA; 3 Department of Emergency Medicine, University of Texas Health, San Antonio, TX 78229, USA; 4 Organ Support & Automation Technology Research Team, JBSA Fort Sam Houston, San Antonio, TX 78234, USA; eric.j.snider3.civ@health.mil (E.J.S.); sofia.i.hernandeztorres.ctr@health.mil (S.I.H.-T.); james.p.collier26.ctr@health.mil (J.P.C.); samantha.k.eaton2.ctr@health.mil (S.K.E.); jose.salinas4.civ@health.mil (J.S.); 5 Biomedical Analytics and Computational Engineering Laboratory, Mayo Clinic, Rochester, MN 55905, USA; holmes.david3@mayo.edu; 6 Special Purpose Processor Development Group, Mayo Clinic, Rochester, MN 55905, USA; haider.clifton@mayo.edu

**Keywords:** lower body negative pressure, hemorrhage, hypovolemia, shock, medical monitoring, wearable sensors

## Abstract

Since hemorrhage is a leading cause of preventable death in both civilian and military settings, the development of advanced decision support monitoring capabilities is necessary to promote improved clinical outcomes. The emergence of lower body negative pressure (LBNP) has provided a bioengineering technology for inducing progressive reductions in central blood volume shown to be accurate as a model for the study of the early compensatory stages of hemorrhage. In this context, the specific aim of this study was to provide for the first time a systematic technical evaluation to meet a commonly accepted engineering standard based on the FDA-recognized Standard for Assessing Credibility of Modeling through Verification and Validation (V&V) for Medical Devices (ASME standard V&V 40) specifically highlighting LBNP as a valuable resource for the safe study of hemorrhage physiology in humans. As an experimental tool, evidence is presented that LBNP is credible, repeatable, and validated as an analog for the study of human hemorrhage physiology compared to actual blood loss. The LBNP tool can promote the testing and development of advanced monitoring algorithms and evaluating wearable sensors with the goal of improving clinical outcomes during use in emergency medical settings.

## 1. Introduction

Hemorrhage remains a major challenge for civilian and military emergency medicine, remaining a leading cause of preventable death. Treatments for managing hemorrhage severity (e.g., development of advanced medical monitoring), as well as developing wearable sensors for early prediction of blood loss and decompensated shock, can improve clinical outcomes in emergency medicine. However, the development of accurate monitoring technologies has suffered from inadequate, costly, or cumbersome experimental models using porcine, dogs, or rats that have not proven to be translatable to humans [1]. Human-based models are needed to study physiological changes during hemorrhage as well as to test and evaluate wearable sensors. Because of the potential adverse effects of removing large volumes of blood from human subjects, a safe procedure in which symptoms associated with progression to a stage of hemodynamic decompensation should be considered [2]. There are more than 20 published articles in the scientific literature in which lower body negative pressure (LBNP) has been used as an experimental model for the study of human hemorrhage [1]. However, there has never been a study in which an engineering standard has been established for LBNP as a hemorrhage model. The specific aim of this study was to provide for the first time a systematic technical evaluation to meet a commonly accepted evidence-based engineering standard with the application of the FDA-recognized Standard for Assessing Credibility of Modeling through Verification and Validation (V&V) for Medical Devices (ASME Standard V&V 40). Our approach highlights a non-invasive LBNP tool as a standardized test platform for the safe study of hemorrhage physiology in humans. The LBNP tool is an experimental system designed to create a vacuum (negative pressure) around the lower part of the body below the waist that results in reduced central blood volume and flow in the upper part of the body. Reducing blood flow to the upper body has been used to simulate the physiology and symptoms during the early compensated phase of blood loss to the onset of hemorrhagic shock in human subjects [1,2,3,4,5,6]. The advantage of the LBNP tool is its safety for human subjects because the vacuum created is an instantaneously reversible technique. The LBNP tool represents a clinical outcome assessment tool because it provides a model that mimics the physiological manifestation of the condition of human hemorrhage. In this paper, we detail the verification and validation of the LBNP procedure as a platform for the study of responses in healthy humans exposed to a surrogate of progressive hemorrhage under safe, ethical, rapidly reversible, and controlled experimental conditions, allowing for the test and evaluation of new monitoring technologies and wearable sensors. Our approach is the first to include a systematic comparison of computational and experimental models using the American Society of Mechanical Engineers standard (ASME-40-2018) for assessment of the credibility assessment of the LBNP through verification and validation application to medical devices [7,8].

## 2. Materials and Methods

### 2.1. Overview of ASME-40-2018

The application of a systematic approach for the development of tools for the test and development of LBNP would represent a new critical standard practice in the medical research community as a support function for the design, testing, and development of medical devices and wearable sensors. Such an approach would ensure, for the first time, the de-risking (i.e., evaluation of safety and performance) of the LBNP tool. The ability to non-invasively reduce central blood volume that mimics the physiology of hemorrhage in healthy humans would provide a powerful tool in biomedical research, augmenting experimental research and technology development through detailed mechanistic and systematic investigations which are not safely available in experiments with other means such as actual blood withdrawal. Thus, human experiments using LBNP to simulate the physiology of hemorrhage are now emerging as an important tool for the testing and development of novel machine learning algorithms, medical monitors, and wearable sensors [1,3,4,5,6].

Biomedical investigators have played a critical role in enabling the use of LBNP with experimental designs and electronic data collection that support the development of advanced medical monitoring technologies. In this regard, one of the most commonly accepted approaches for the establishment of the LBNP as a human hemorrhage tool is to apply the FDA-recognized Standard for Assessing Credibility of Computational Modeling through Verification and Validation (V&V) of Medical Devices specifically to the proposed LBNP tool (ASME standard V&V 40). While this standard is meant to validate computational models [7,8], we took a novel approach to demonstrate how its principles can be applied to experimental platforms such as LBNP.

**Figure 1:**
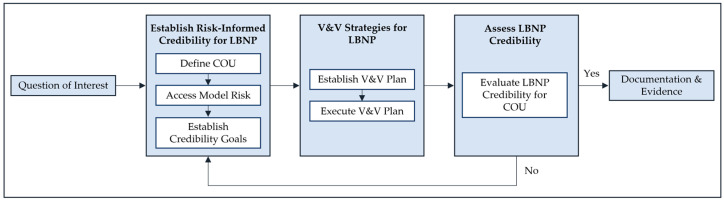
The risk-informed credibility assessment framework of ASME V&V40 for the LBNP tool. Reprinted/adapted from References [7,8] with permission from the American Society of Mechanical Engineers (Copyright year 2018). All rights reserved.

Briefly, the standard begins by defining the question of interest for the intended experimental or computation model and establishes the risks associated with the model (Figure 1). Next, model credibility is evaluated, for which we determined a set of requirements to demonstrate that LBNP has sufficient credibility for the context of use. To assist in this process, the ASME V&V 40 standard defines a set of credibility factors, which represent the constituent elements of a credibility evaluation process. In this paper, we systematically provide for the first time evidence regarding the accuracy of each of these factors in order to demonstrate that the overall credibility of LBNP as a surrogate for ongoing hemorrhage is commensurate with our determined model risk.

The last element of the ASME standard is applicability, for which we detail the development of a computer model for tracking the progressive reduction in central blood volume known as the compensatory reserve measurement (CRM). The collection of a CRM generated specifically from the proposed LBNP protocol was subsequently used as a method for assessing the state of compensation during progressive reductions in central circulating volume (i.e., central hypovolemia). The use of LBNP provided a novel and safe approach to inducing central hypovolemia that is associated with actual hemorrhage for the test and evaluation of medical devices and wearable sensors designed to track reductions in central blood volume.

### 2.2. Overview of Lower Body Negative Pressure for Simulation of Hemorrhage

As an experimental platform, LBNP has been used to provide a consistent measure of the progression of reduced central blood volume (i.e., central hypovolemia) in humans with utility for use in the performance analysis of medical devices and wearable sensors that are proposed to detect the presence and progression of hemorrhage. Specifically, LBNP could be used as a platform for testing any monitoring technologies or sensors that provide the capability to measure heart rate, stroke volume, cardiac output and contractility, arterial blood pressure, arterial pulse pressure, central venous pressure, peripheral vascular resistance, blood acidity, blood lactate, blood base excess, blood partial pressure of oxygen, blood partial pressure of carbon dioxide, blood glucose, sodium and osmolality in the plasma, white blood cells, platelet counts, and clotting factors because LBNP has been shown to accurately mimic these physiological responses when compared directly to actual blood loss [1,2,3,5]. LBNP provides an ability to test for the safety or effectiveness of a given diagnostic or therapeutic device in achieving its intended clinical effect. For instance, the United States Department of Defense (DOD) has created a consortium focused on the development of non-invasive technologies for early diagnosis and provider alert of decompensation due to hemorrhage and hemorrhagic shock [9]. Such assessments can be used to inform earlier lifesaving interventions and improve patient outcomes. The LBNP tool could provide the DOD and the civilian emergency medical community with the capability of evaluating the head-to-head performance of various proposed monitoring and detection devices.

**Figure 2:**
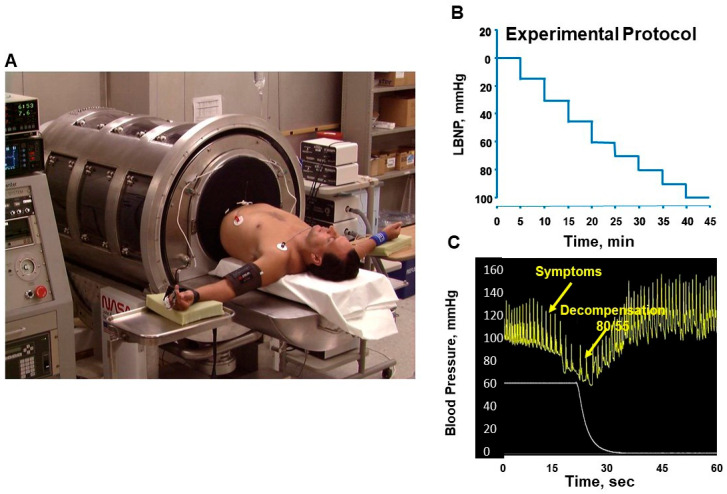
Human subject undergoing lower body negative pressure (LBNP, (**A**)). A standard experimental protocol consists of stepwise reductions in pressure inside the chamber (**B**). The protocol is terminated with a precipitous fall in systolic blood pressure to <80 mmHg accompanied by concurrent subjective symptoms (e.g., dizziness, loss of peripheral vision, disorientation, impaired responsiveness) (**C**). Releasing the negative pressure in the chamber returns central blood volume and pressure to normal, optimizing safety for the subject (**C**). Modified from References [4,7].

The LBNP tool consists of 3 elements: an LBNP chamber, a vacuum pressure control system, and a specific pressure profile protocol. Subjects are positioned supine with their legs within a chamber that is sealed airtight at the waist (level of the iliac crest) using a neoprene skirt (Figure 2A). The pressure in the chamber can be controlled with profiles of negative pressures (Figure 2B) in a fashion that depicts progressive hypovolemia similar to that produced by actual hemorrhage. The increasing negative pressure causes a progressive redistribution of blood away from vital organs of the upper body circulation (e.g., heart, brain) to the lower body below the waist. A unique advantage of this experimental approach is that the clinical tolerance of each individual can be assessed by creating a reduced central blood volume at which physiological mechanisms of compensation can no longer accommodate the requirement for adequate blood flow and oxygen delivery to the brain, leading to hemodynamic instability and decompensation (Figure 2C).

## 3. Results

### 3.1. V&V 40-2018: Context of Use

#### 3.1.1. Question of Interest

Is the LBNP model a suitable non-invasive model of human hemorrhage for developing advanced monitoring algorithms and testing novel sensors? The LBNP functions as a replacement for the testing and evaluation of technologies required for monitoring of human hemorrhage in which it would not be ethical or feasible if significant blood withdrawal were required. LBNP also provides a method of evaluation for device technologies that are historically evaluated through other bench or animal testing that may not be translatable to conditions of human blood loss. Additionally, LBNP can serve as a platform for demonstrating safety, effectiveness, or device performance in a clinical setting.

#### 3.1.2. Context of Use

The LBNP tool serves as a valid platform for the testing and evaluation of physiological (i.e., clinical) signals or devices (e.g., algorithms, wearable sensors) that detect compensation due to blood loss during the early compensatory phase of circulatory shock. This instrument is considered a clinical outcome assessment, clinician-reported outcome per the FDA’s Medical Device Development Tool program. The LBNP tool gives researchers and clinicians the ability to utilize a safe, stepwise process in gradually reducing an individual’s central blood volume (i.e., central hypovolemia). Use of the LBNP model is limited to healthy individuals aged between 18 and 65 years who have been screened with both a physical examination and a thorough review of their medical history. This tool provides device developers with the ability to evaluate their hemorrhage detection technologies in early clinical studies.

### 3.2. V&V 40-2018: Model Risk Assessment for LBNP

Model risk is generally defined as a combination of the influence and decision consequence in using the LBNP. For the LBNP tool, influence “represents the contribution of the model to the decision in relation to other available evidence” [8]. Model influence for LBNP was considered by our assessment to be ‘low’ as the maximal vacuum level reached by the chamber for any experiment is not used as a means of measuring experiment completion. Instead, patient physiology, assessment, and feedback from the LBNP subject are the basis for stopping LBNP regardless of what pressure step the experiment has reached.

Decision consequence refers to “the significance of an adverse outcome resulting from an incorrect decision (i.e., the “severity” of the adverse outcome if the decision based on the model is incorrect” [8]. For the LBNP tool, ‘decision consequence’ can be more impactful as excessive negative pressure and subsequent hypovolemic levels can lead to a patient passing out. There has never been an adverse event from an LBNP experiment conducted in the U.S. Army Institute of Surgical Research (USAISR) laboratory using healthy male and female volunteers as subjects within the age range of 18 to 65 years who have been screened with both a physical examination and medical history. In addition, published observations indicate that LBNP can be well tolerated in healthy elderly men and women ranging in age from 68 to 74 years [1].

Several safety measures have been implemented to ensure the protection of the health, safety, and wellbeing of human volunteers. An emergency button has been implemented that can initiate immediate shut-off of the vacuum and open the valve to the chamber for maximum equilibration of pressure if any issue is noted. A second safety feature is the capability to use a manual bleeder valve to open the entire system and subsequently vent the chamber, resulting in an instant release of the negative chamber pressure. In the case of the LBNP system located at the USAISR, a computer program that controls the LBNP vacuum profile has a threshold set such that any pressures outside of the prescribed ±2 mmHg at each level will automatically shut off the system. Finally, the LBNP system can be manually shut down, resulting in the immediate opening of the valve and release of the vacuum, quickly returning central blood volume to normal baseline values. Should the subject ever feel uncomfortable or want to stop, the LBNP can be shut off using a manual stop.

For this reason, we rate the LBNP as having a low model influence with medium decision consequence, resulting in an overall medium risk for the LBNP tool.

### 3.3. V&V 40-2018: Model Credibility—Verification of LBNP

Having identified the overall LBNP tool risk as medium, a strategy was introduced to establish the credibility requirements for the LBNP. As defined by ASME V&V 40-2018 [7], LBNP credibility refers to the trust in the predictive capability of LBNP for our specific context of use, where we can establish trust through the collection of verification and validation (V&V) evidence and by demonstrating the applicability of those V&V activities to support the use of LBNP for our context of use (test and evaluation of medical devices and wearable sensors).

The protocol used to support the verification of LBNP as a non-invasive model of human hemorrhage for the test and evaluation of medical devices and wearable sensors consisted of a 5 min control rest period (0 mmHg LBNP) to establish physiological baseline values followed by 5 min of chamber decompression to levels of −15, −30, −45, and −60 mmHg and additional increments of −10 mmHg every 5 min until the onset of hemodynamic decompensation (i.e., threshold for decompensated shock).

To verify the operating procedures required to operate the LBNP vacuum chamber were working accurately, periodic testing of the machine setup was conducted without a human subject. The chamber can be sealed to allow the full protocol through 5 min at −100 mmHg, and the output chamber pressures can be recorded to ensure the accuracy of the pressure within a prescribed tolerance level of less than ±2 mmHg. Importantly, the vacuum pump controller is set such that anytime the pressure exceeds a set tolerance limit, the pressure will shut off to ensure subject safety. This verification procedure ensures that the protocol is executed at the desired levels of LBNP. Table 1 and Table 2 detail the recorded pressure readings from a full protocol run both with and without a human subject, demonstrating the pressure readings to be within the desired tolerance threshold at each prescribed level of LBNP.

Hemodynamic decompensation can be identified in real time as a precipitous fall in systolic blood pressure >15 mmHg within 5 to 10 s, or progressive diminution of systolic blood pressure to less than 80 mmHg, and/or the presence of symptoms expressed by the subject such as gray-out, sweating, nausea, or dizziness. These thresholds of systolic blood pressure and/or mental status were used to identify the stage and time for terminating LBNP based on clinical practice guideline doctrine [10]. An additional analysis that can be performed to demonstrate credibility for LBNP as a human hemorrhage model is through the calculation of test–retest correlation coefficients. As such, high inter-subject reproducibility with durations between repeat LBNP tests in the same subjects at 30 min, 2 weeks, and 1 year, each having correlation coefficients ≥ 0.821 (Table 3).

### 3.4. V&V 40-2018: Model Credibility—Validation of LBNP

In order to ensure that LBNP provides a valid representation of progressive hemorrhage for the test and evaluation of medical devices and/or wearable sensors, as well as quantify the errors present in the use of LBNP, we present experimental evidence generated from a direct comparison of hemodynamic responses generated from experimentally controlled hemorrhage and LBNP.

Consistent with the context of use that the LBNP serves as a platform for generating integrated responses to hemorrhage that are required for accurate testing and evaluation of devices and/or wearable sensors, we compared physiological outputs generated from LBNP and the validation comparator of actual hemorrhage in non-human primates. The validation metric was a mathematical demonstration of statistical similarity in these physiological responses between LBNP and the comparator (actual progressive hemorrhage).

**Figure 3:**
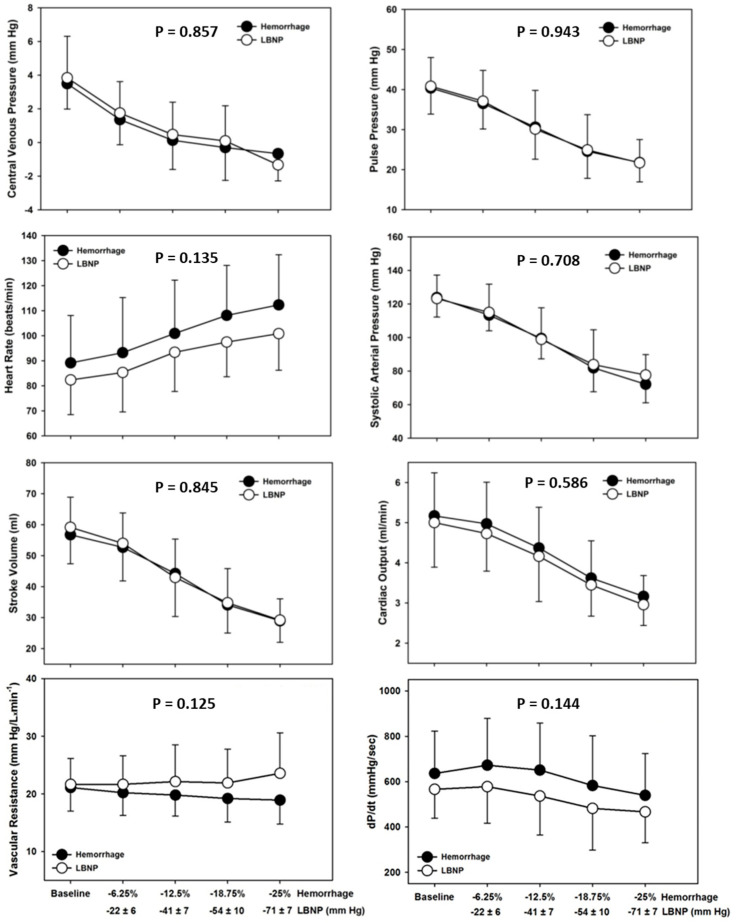
Central venous pressure, pulse pressure, systolic blood pressure, heart rate, stroke volume, cardiac output, vascular resistance, and cardiac contractility (+dP/dt) during baseline and 4 steps of hemorrhage (closed circles) and lower body negative pressure (LBNP; open circles) corresponding to 6.25% (*n* = 14), 12.5% (*n* = 14), 18.75% (*n* = 14), and 25% (*n* = 12) total blood volume loss. Data are expressed as means ± SD. Two-factor repeated-measures ANOVA *p*-values are shown for differences between hemorrhage and LBNP. Modified from Hinojosa-Laborde et al. [13].

For validation testing, the data obtained from twelve adult male baboons (age = 8 to 12 years; body weight = 25–35 kg) were used to compare hemodynamic responses to hemorrhage and LBNP [13]. The subjects were sedated and intubated to maintain an open airway that allowed for spontaneous breathing. Direct continuous analog recordings of hemodynamic signals were obtained invasively from an axillary arterial catheter. For the hemorrhage experiment, blood was withdrawn in four steps of hemorrhage that approximated 6.25% (4.5 mL/kg), 12.5% (9.1 mL/kg), 18.75% (13.7 mL/kg), and 25% (17.7 mL/kg) blood volume estimated by body weight. Each step was 7 min in duration. Four weeks after the hemorrhage experiment, the same baboons were again sedated and instrumented for recording analog arterial pressure waveforms and placed supine in an LBNP chamber. LBNP levels for each step (7 min each) were determined by matching central venous pressure (measured from a catheter placed in the right atrium) from the animal’s previous hemorrhage study. The recorded hemodynamic analog signals from both hemorrhage and LBNP experiments were then used for statistical comparisons. The results supported the notion that LBNP is a valid tool for reproducing the physiology of hemorrhage required for accurate testing and evaluation of medical devices and wearable technologies since the hemodynamic values recorded during baseline and the four steps of hemorrhage and LBNP were statistically indistinguishable from one another. The four levels of blood volume withdrawal translated to average LBNP levels of approximately 20, 40, 50, and 70 mmHg LBNP (Figure 3).

### 3.5. V&V 40-2018: Model Credibility—Applicability of LBNP

The ASME standard V&V 40 has been generally used by medical device manufacturers that are seeking FDA 510k clearance to validate software components of medical devices. Within this context, a machine learning algorithm software component was used to test the model credibility of the LBNP for tracking blood loss volume. This approach was recommended to be included as part of a prequalification submission for certification of LBNP as a non-invasive human hemorrhage model under the FDA Medical Device Development Tool program. Three steps of credibility for the applicability of LBNP as a tool that serves as a valid platform for the testing and evaluation of physiological signals or devices that accurately reflect the physiology of human hemorrhage are presented in this paper. We first describe the rationale for translating reductions in central blood volume induced by LBNP to the development of the CRM algorithm. We then demonstrate the quantification of reductions in CRM with reductions in LBNP as a surrogate of central hypovolemia. Finally, we use CRM developed with LBNP data sets to demonstrate the superiority of a technology derived from LBNP over standard-of-care vital sign measurements in patients suffering from clinical conditions of low circulating blood volume.

#### 3.5.1. Overview of Compensatory Reserve Measurement Algorithm

Computational algorithms have been developed from LBNP datasets for tracking patient physiology. Physiological data were generated from experiments conducted at the USAISR on 201 human subjects using LBNP [6]. From this data set, a machine learning algorithm was developed to provide a measurement of the integrated physiological reserve to compensate for clinical conditions of low circulating blood volume (CRM). The algorithm is FDA-cleared (DEN160020) with the indication that it “trends changes in intravascular volume relative to the individual patient’s response to hypovolemia”.

The functional framework for the CRM algorithm is based on changes in the morphology of arterial waveform features [3,4,5,13]. From our human LBNP experiments, a software framework was applied to a library of >650,000 analog arterial waveform recordings. The computational algorithm for tracking the CRM was developed from the application of a one-dimensional convolutional neural network time series data regression structure [14]. This approach led to the interrogation of hundreds of features within each non-invasive arterial waveform that trend the compensatory phase of central blood volume loss. In this regard, the algorithm was constructed with the use of the following generalized equation to calculate an estimate of CRM:

$$\mathrm{CRM}=[1-\frac{BLV}{BLV@HD}]\times \;100$$

where BLV is the current blood loss volume of the human subject, and BLV@HD is the blood loss volume at which the onset of hemodynamic decompensation occurs in that subject. Within this construct, the calculated estimate of CRM relied on the assumption that an individual’s blood loss volume at any given time is known, as well as that individual’s BLV@HD due to reduced central blood volume. The accuracy of this assumption is supported by experiments using non-human primates and human subjects that demonstrated how LBNP closely mimics physiologic and compensatory responses observed when compared to hemorrhage [1,5,13,15]. These direct comparisons allowed for the translation of −30, −60, and −90 mmHg LBNP to average equivalents of approximately 450, 1000, and 1600 mL blood loss in a 70 kg human [13]. As such, the relationship between LBNP and BLV allowed for an ethically and scientifically justified substitute for modeling the reduction in central blood volume to a physiological outcome of hemodynamic decompensation in humans using the following calculation to estimate CRM:

$$\mathrm{CRM}=[1-\frac{BLV(t)}{BLV@HD}]\times \;100\;\approx \;[1-\frac{LBNP(t)}{LBNP@HD}]\times \;100$$

where LBNP(t) is the LBNP level that the individual is experiencing at time t, and LBNP@HD is the LBNP level at which there is an onset of hemodynamic decompensation in that individual. As such, the CRM was modeled as either a linear function over the duration of the LBNP experiment or as a series of steps corresponding to the applied LBNP, with LBNP level used as a target of reduced central circulating blood volume.

#### 3.5.2. Strength of Evidence for Tracking Blood Loss Volume during LBNP in Humans

A statistical analysis using generalized estimating equations was used for calculating the receiver operating characteristic area under curve (ROCAUC) for sensitivity and specificity of the CRM algorithm across 191 subjects during progressive stepwise reductions in central blood volume (i.e., tracking LBNP levels). The ROC AUC for detection of central blood volume reduction (i.e., central hypovolemia) and prediction for the onset of decompensated shock was 0.9411 (0.0059) with 95% confidence limits of 0.9299 to 0.9523 (*p* = 0.0103) for the CRM. A ROCAUC value of >0.9 is indicative of a model being highly accurate [16]. Within this reference, the CRM algorithm displayed a higher accuracy rating for detecting reduced circulating blood volume and predicting the onset of decompensated shock using the LBNP protocol presented in the paper compared to moderately accurate (0.7 < ROCAUC ≥ 0.9) standard vital signs such as systolic blood pressure (0.81), heart rate (0.73), perfusion index (0.56), pulse pressure variability (0.79), respiratory rate (0.51), blood lactate (0.54), tissue oxygen saturation (0.68), end-tidal CO_2_ (0.70), and various measures of heart rate variability and complexity (0.70 ≤ ROCAUC ≥ 0.84) [4].

**Figure 4:**
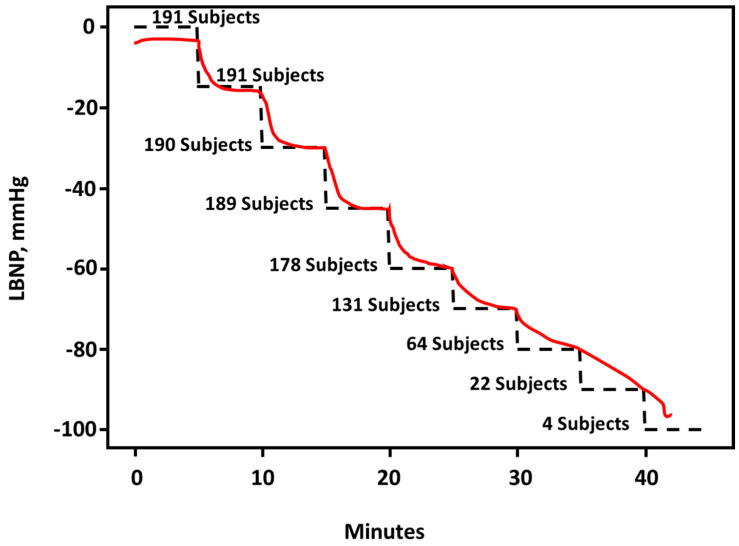
Average responses of compensatory reserve during stepwise LBNP in 191 subjects estimated by the CRM algorithm for predicting reductions in central circulating blood volume and decompensated shock. Data extracted from Techentin et al. [14].

A generalized linear mixed statistical analysis was used to determine average CRM responses that tracked LBNP levels across 191 subjects during progressive stepwise reductions in central blood volume (Figure 4). The number of subjects who progressed through each stage of LBNP is also presented.

**Figure 5:**
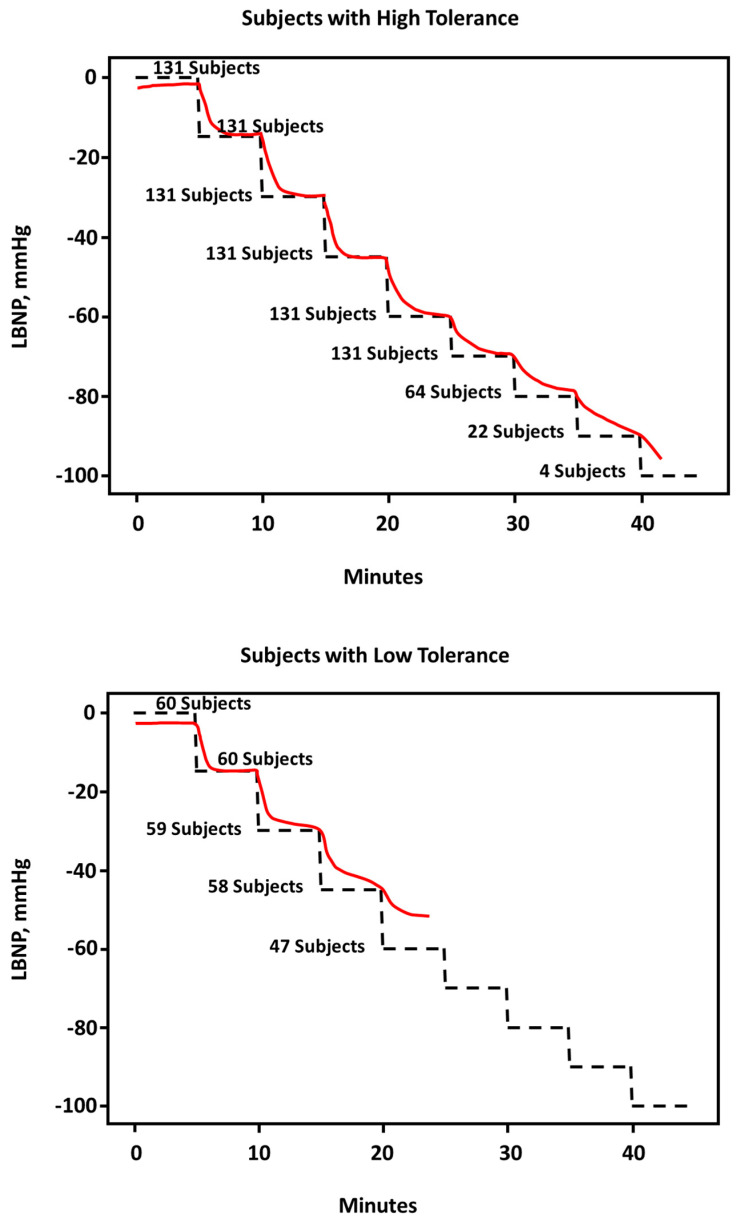
Average responses of compensatory reserve during stepwise LBNP estimated by the CRM algorithm for predicting reductions in central circulating blood volume and decompensated shock for 131 high tolerant subjects (**upper panel**) and 60 low tolerant subjects (**lower panel**). Data extracted from Techentin et al. [14].

Subsequently, the 191 participants were categorized as those with high tolerance (*n* = 131) or low tolerance (*n* = 60) to reductions in circulating central blood volume (i.e., central hypovolemia) using previous statistical analysis of Kaplan–Meier “survival” curves [5]. By definition, participants with low tolerance experienced the onset of decompensated shock prior to completing an LBNP level of −60 mmHg (total protocol time < 1500 s including baseline rest), while participants with low tolerance completed LBNP levels > −60 mmHg (>1500 s of the total protocol). Using the same statistical approach as that used for all 191 subjects, calculations of CRM values were based on changes in arterial waveform analog features collected non-invasively from all subjects (i.e., there were no CRM values constructed for separate prediction of decompensated shock in high and low tolerance subjects). CRM responses were analyzed based on tolerance classification, an approach that accurately tracked the reductions in targeted central blood volume (i.e., LBNP level) in both high tolerance subjects (Figure 5, upper panel) and low tolerance subjects (Figure 5, lower panel) independent of one another. These results demonstrated validation of using CRM as an accurate assessment of reductions in central blood volume induced by LBNP across individuals with differing compensatory capacities.

#### 3.5.3. Clinical Evidence for Compensatory Reserve Measurement

The target application for CRM is the clinical setting of patients who undergo conditions of reduced circulating blood volume resulting from hemorrhage or sepsis. Within this context, our approach to address the applicability of LBNP for its ability to track changes in hypovolemia was to assess the CRM in representative clinical cohorts of such patients with controlled or uncontrolled bleeding.

Evaluation of the applicability of an arterial waveform analysis metric such as CRM can be addressed through a systemic literature review of clinical investigations that include AWFA in patients experiencing changes in circulating blood volume status [17,18,19,20,21,22,23,24,25]. Table 4 provides a comparison of sensitivity and specificity characteristics of arterial waveform analysis with various standard vital signs as measured with ROC AUC analysis across seven clinical studies. In all cases, the ROCAUC was clinically higher using arterial waveform analysis than any currently available standard vital sign. These results support the clinical significance of translating some form of arterial waveform analysis (e.g., CRM) generated from LBNP as a valid metric for earlier and individualized assessment of medical devices and/or wearable sensors, particularly in an emergency medical scenario when time is of the essence in promoting ‘good’ clinical outcome. Arterial waveform analysis is feasible with LBNP studies as non-invasive methods using photoplethysmography have successfully been used to generate arterial waveforms during negative pressure experiments [1,3,4].

## 4. Discussion

The need to develop a validated model of hemorrhage and hypovolemia remains critically important as the pace of development for advanced decision support monitoring devices and new wearable sensors for hemorrhage detection is accelerated. Coupling with the integration of advanced artificial intelligence algorithms that have the potential for continuously evolving during the deployment of such technologies will require a re-evaluation of experimental approaches currently used for device testing and validation. However, evaluation of these types of devices specifically designed for measuring and/or detecting levels of hemorrhage is limited by the inherent compensatory mechanisms that mask true injury severity in many trauma/critical care environments, in addition to other environmental factors associated with human trials in critical care settings. The results presented in this paper address this specific gap in the field by providing verification and validation of LBNP as a safe surrogate for progressive blood loss in humans for the development and evaluation of advanced monitoring algorithms and wearable sensors.

The use of the LBNP model has proven to be a valuable tool in the detection of hemorrhage, offering several notable benefits in both research and clinical settings. Based on the results of the present investigation, LBNP has been shown to provide a standardized way to simulate hemorrhage without the need for actual blood loss in humans. This is particularly advantageous in device development, as it allows researchers to study the physiological responses to hemorrhage in a laboratory-controlled environment and, therefore, enables accurate validation of wearable sensors. The ability of the LBNP to adjust through varying negative pressure values provides a novel capability to test different levels of hemorrhage severity, which would be a challenge to achieve in standard device trials.

Further, the LBNP offers a safe and non-invasive means of assessing an individual’s physiological response to hemorrhage. By subjecting human participants to specifically controlled negative pressure, clinicians and researchers can observe how the body’s compensatory mechanisms, such as elevated heart rate and peripheral vasoconstriction, respond to reduced central blood volume similar to that associated with actual hemorrhage. This insight is particularly valuable for early detection of ongoing blood loss; subtle clinical changes that may not be immediately apparent through traditional clinical assessments that depend on standard or traditional vital signs can be identified in the early stages of hemorrhage.

LBNP can also serve as a training tool for healthcare professionals. Medical personnel can gain experience in recognizing and managing hemorrhage-related conditions, improving their ability to respond effectively in real-life situations. By allowing for repeated practice in a controlled environment, LBNP can contribute to enhancing the skills and confidence of medical practitioners, ultimately leading to better patient outcomes in cases of hemorrhage and other clinical conditions of severe hypovolemia.

## 5. Limitations

Although ethical and practical challenges with the implementation of LBNP in healthy subjects should not be ignored, an extensive review of subject recruitment, consent, safety, and compliance has revealed the existence of no such issues [2]. Given that the V&V assessment presented in this study was conducted in healthy, non-injured humans, we cannot dismiss potential confounding clinical impacts without addressing the possibility that factors common to trauma, such as inflammatory responses, coagulopathy, hypothermia, pain, or severity of tissue injury may affect the validity and generalizability of LBNP as a hemorrhage model. Contrary to the possibility that the validity of LBNP as a hemorrhage model may be influenced by various uncontrolled factors associated with injury or disease states, clinical data consistently reported in the literature across multiple clinical studies (see Table 4) with inclusion of patients with various ages and genders support the notion that there appears to be negligible impact on medical monitoring technologies that have been generated from the use of LBNP [1,9]. The potential contribution of genetic factors related to the physiology of high and low tolerance to blood loss awaits the results from currently ongoing investigations in our laboratory in which multi-omic measurements are being conducted.

## 6. Conclusions

Through the ASME-40-2018 verification and validation protocol, we have shown that LBNP is a credible model for a context of use focused on non-invasive hemorrhage modeling. We first established that LBNP poses a medium risk to the end user as the decision consequence can be severe, but the model influence is minimal as LBNP settings are not used to dictate the experiment outcome. Instead, patient physiology drives the experiment endpoint. In addition, extensive safety measures are present in LBNP to ensure the pressure is precisely controlled and normal pressure can be rapidly restored if pressure settings deviate from expected values. The novelty of our present study lies in demonstrating for the first time the validation of the LBNP using the ASME-40-2018. This approach allowed LBNP to be tested with an industry-supported standard that provides researchers and medical device developers with advanced knowledge and understanding of a tool designed to test new hemorrhage detection devices in an environment that has been shown to mimic the physiologic responses of real hemorrhage, but in a standardized and controlled model that can also be replicated and adjusted as needed.

Through credibility activities, we first demonstrated LBNP verification through confirmation of pressure setpoint holding at correct values with minimal standard deviation. This is critical from an experimental repeatability standpoint and is further shown by verification activities involving re-testing subjects through LBNP. No matter if the test was repeated after only 30 min or up to one year later, the reproducibility of physiological responses is supported by strong correlation coefficients. Validation activities compared hemorrhage and LBNP results in non-human primates, for which a number of key vital signs were tracked similarly between the two procedures. This confirms that the volume re-distribution occurring as a result of negative pressure applied to the lower body is analogous to actual blood loss. Lastly, the applicability of LBNP was highlighted through the development and testing of the compensatory reserve measurement algorithm. Advanced monitoring technologies such as CRM have proven ideal for development using LBNP training data sets. The CRM has subsequently been FDA-approved and clinically validated in various medical applications, further highlighting the relevance of LBNP as a standardized test platform for evaluating hemorrhage in human subjects.

## Figures and Tables

**Table 1:** LBNP pressure readings without a human subject through the full step protocol.

Set Pressure Profile (mmHg)	Average Pressure Reading (mmHg)	Standard Deviation Pressure Reading (mmHg)
−15	−15.07	±1.62
−30	−30.12	±1.66
−45	−45.15	±1.75
−60	−60.16	±1.75
−70	−70.17	±1.78
−80	−80.16	±1.82
−90	−90.16	±1.86
−100	−100.16	±1.91

**Table 2:** LBNP pressure readings with a human subject through the full step protocol.

Set Pressure Profile (mmHg)	Average Pressure Reading (mmHg)	Standard Deviation Pressure Reading (mmHg)
−15	−14.83	±1.62
−30	−29.83	±1.66
−45	−44.83	±1.71
−60	−59.83	±1.74
−70	−69.84	±1.77
−80	−79.82	±1.81
−90	−89.82	±1.84
−100	−99.82	±1.88

**Table 3:** Test–retest correlation coefficients for the testing of LBNP tolerance repeatability in human subjects. Data summarized from Convertino et al. [11,12].

Time between LBNP Procedures	N	Correlation Coefficient (r)	Reference
30 min	7	0.821	Convertino and Sather [11]
2 weeks	11	0.914	Convertino and Sather [11]
1 year	7	0.914	Convertino [12]

**Table 4:** Comparisons of ROC AUC for assessment of sensitivity and specificity in various clinical conditions using models of CRM compared to various standard vital signs. Modified from Convertino and Cardin, Ref. [9]. AWFA, arterial waveform feature analysis; SBP, systolic blood pressure; HR, heart rate; PP, pulse pressure; SI, shock index; Lac, blood lactate.

Reference	N	Clinical Condition	AWFA	SBP	HR	PP	SI	Lac
Nadler er al [18]	230	Blood Donation	0.84	0.60	0.73	0.51	0.64	–
Stewart et al. [19]	122	Blood Donation	0.90	0.84	0.55	–	–	–
Convertino et al. [20]	20	Controlled Hemorrhage	0.90	0.62	0.59	0.72	0.72	–
Mackenzie et al. [21]	556	Trauma Hemorrhage	0.78–0.89	–	0.56–0.62	–	–	–
Stewart et al. [22]	44	Trauma Hemorrhage	0.97	0.81	0.64	–	0.74	0.73
Benov et al. [23]	31	GI Bleeding	0.79	0.62	0.60	0.36	–	–
Johnson et al. [24]	89	Trauma Hemorrhage	0.83	0.62	–	–	–	–
Benov et al. [25]	100	Sepsis	1.00	0.67	0.78	0.56	–	–

## Data Availability

The data presented in this study are not publicly available because they have been collected and maintained in a government-controlled database that is located at the U.S. Army Institute of Surgical Research. As such, these data can be made available through the development of a Cooperative Research and Development Agreement (CRADA) with the corresponding author.
